# Socioeconomic status and delayed surgery: impact on non-metastatic papillary thyroid carcinoma outcomes

**DOI:** 10.3389/fpubh.2024.1488294

**Published:** 2025-01-22

**Authors:** Kun Zhang, Xinyi Wang, Jianyong Lei, Anping Su, Tao Wei, Zhihui Li, Ya-Wen Chen

**Affiliations:** ^1^Division of Thyroid Surgery, Department of General Surgery, West China Hospital, Sichuan University, Chengdu, Sichuan, China; ^2^Department of Otolaryngology, Icahn School of Medicine at Mount Sinai, New York, NY, United States; ^3^Department of Cell, Developmental, and Regenerative Biology, Icahn School of Medicine at Mount Sinai, New York, NY, United States; ^4^Black Family Stem Cell Institute, Icahn School of Medicine at Mount Sinai, New York, NY, United States; ^5^Institute for Airway Sciences, Icahn School of Medicine at Mount Sinai, New York, NY, United States; ^6^Center for Epithelial and Airway Biology and Regeneration, Icahn School of Medicine at Mount Sinai, New York, NY, United States

**Keywords:** overall survival, prognostic factors, papillary thyroid cancer, socioeconomic status, surgery delay

## Abstract

**Introduction:**

The growing popularity of active surveillance for papillary thyroid cancer and the COVID-19 pandemic have increased surgery delay, further necessitating a reassessment of the link between surgery delay and survival outcomes for papillary thyroid cancer. In this study, we aim to investigate the interplay among various oncological factors, socioeconomic status, and surgical timing with respect to survival outcomes of papillary thyroid cancer.

**Methods:**

A total of 58,378 non-metastatic papillary thyroid cancer patients from 2000 to 2018 were screened from the Surveillance, Epidemiology, and End Results database. Kaplan–Meier survival curve, Cox proportional hazard regression, competing risk hazard regression, and multinomial logistic regression were applied.

**Results:**

Receiving neck dissection or radioactive iodine therapy, being married at diagnosis, living in an urban area, being richer, and being of other minority ethnicity were estimated to be independent predictors for better overall survival. Single, older Black patients living in rural areas that experienced long surgery delays were more associated with a higher non- papillary thyroid cancer mortality rate. High income level was the only independent socioeconomic status predictor for lower papillary thyroid cancer -specific mortality. Unmarried, older patients of minority ethnicity tended to undergo longer surgery delays.

**Conclusion:**

Surgery for non-metastatic papillary thyroid cancer patients can be safely delayed. The elevated non-papillary thyroid cancer mortality has reflected low socioeconomic status population’s survival status.

## Introduction

Health care disparities including state of disease, medical intervention, timing of therapy and socioeconomic status (SES) can greatly affect patient outcomes in a wide variety of diseases (1–4). Data pertaining to the association between SES and cancer mortality have been clearly shown in breast cancer (5), lung cancer (6), colorectal cancer (6), and oropharyngeal cancer (7). Patients with higher SES commonly have a better chance of early-stage diagnosis, resulting in better outcomes (8). However, despite the fact that prognostic factors for thyroid carcinoma have been well established (9), how SES affects its survival outcomes has not been well described. For PTC, it is observed that individuals with better access to healthcare tend to be over-diagnosed with early-stage disease (10, 11). There is also evidence associating PTC patients with lower SES with worse survival outcomes (12–15). Though previous studies have examined the effect of time-to-surgery on PTC (16, 17), the interplay between oncological factors, SES, and surgical timing on survival outcomes has yet to be fully explored (16, 17). Our data could potentially provide some supporting evidence to advance that endeavor.

In this study, we acknowledge the complex interplay between SES and time to surgery, recognizing that time to surgery may function as a mediator influencing patient outcomes, rather than solely as a confounder or interaction variable. This perspective aligns with principles of mediation analysis, suggesting that SES can indirectly affect outcomes through its impact on time to surgery (18, 19). Although we did not perform a formal mediation analysis, we opted to treat surgery delay as the independent variable because it is more directly measurable and clinically actionable, unlike SES, which is not routinely captured in clinical practice.

Our study seeks to evaluate the impact of surgery delay on the survival of PTC patients, while considering the potential influence of SES on PTC incidence and mortality, among other contributing factors. Using the SEER database, we analyze how sociodemographic variables and surgery delay affect patient outcomes, offering valuable insights into their individual and combined effects.

## Materials and methods

### Data sources

The study acquired a cohort of pathologically confirmed PTC patients from the SEER program (20). The study was reported in accordance with the guidelines outlined in the STROBE statement (21). The selected database, in accordance with our previous studies (22, 23), is cited as: “Incidence - SEER Research Plus Data, 18 Registries, Nov 2020 Sub (2000–2018) - Linked To County Attributes - Total U.S., 1969–2019 Counties, National Cancer Institute, DCCPS, Surveillance Research Program, released April 2021, based on the November 2020 submission.” As mandated by the SEER program (24), cancer patient follow-up is conducted by both hospital-based and many population-based registries. These registries employ similar procedures to ensure ongoing medical surveillance, enabling the determination of treatment outcomes and the monitoring of the health status of the cancer population.

### Study cohort selection

**Figure 1:**
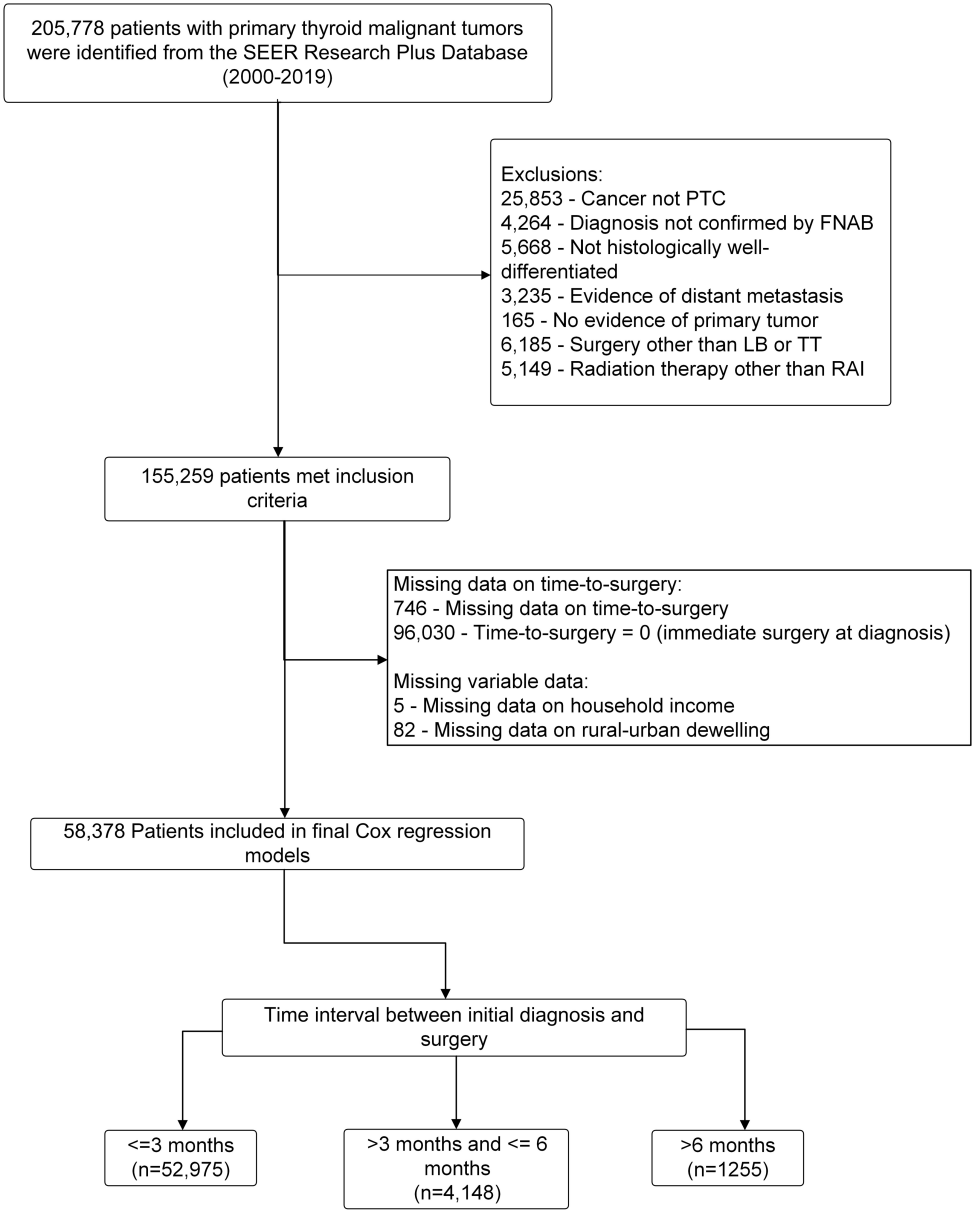
The workflow of non-metastatic PTC patient screening.

This retrospective cohort study initially included 205,778 consecutive patients with pathologically confirmed thyroid malignancies who underwent initial thyroid surgery between 2000 and 2018 from the SEER registry described above. The study established the cohort by excluding patients who met any of the following criteria: (1) their cancer was not PTC; (2) their diagnosis had not been confirmed through fine needle aspirational biopsy (FNAB); (3) their cancer was not histologically well-differentiated; (4) they showed evidence of distant metastasis; (5) they had undergone a surgical procedure other than lobectomy (LB) or total thyroidectomy (TT), or had not undergone surgery at all; and (6) they had undergone radiotherapy other than radioactive iodine. A total of 155,259 FNAB confirmed PTC patients with no evidence of distant metastasis were selected by this exclusion criteria. Furthermore, we excluded a total of 96,030 patients who underwent immediate surgery (time-to-surgery = 0), 764 patients who had missing information regarding their time-to-surgery, 82 patients who lacked information about their rural–urban dwelling environmental setting, and 5 patients who had missing data on their household income. These exclusions ensured that the study sample was appropriate for our research aim by defining pre-surgical FANB-confirmed PTC patients with no evidence of distant metastasis who did not undergo immediate surgery. Participants who underwent immediate surgery (*n* = 96,030) were excluded to ensure the study specifically focused on patients with a documented delay in surgery, as this aligns with the research objective of analyzing the impact of delayed surgery on outcomes. In total, 58,378 eligible non-metastatic PTC patients that did not receive immediate thyroid surgery were included in the final Cox regression model with subgroups divided by time of surgery delay: (1) ≤3 months (which served as the reference value); (2) >3 months and ≤6 months; and (3) >6 months. The workflow of the cohort selection is illustrated in Figure 1.

### Study variables

Our study extracted relevant variables for statistical analysis, including demographics, tumor staging, SES and therapeutic approaches: age, gender, race, primary site of tumor, pathology, AJCC (American Joint Committee on Cancer) stage, SEER stage (25), marital status at diagnosis, median household income, rural–urban dwelling environmental setting, primary surgery, neck dissection, radiotherapy, chemotherapy, systemic therapy, survival months, cause of death, and survival status.

### Outcome definition

The study aimed to assess overall survival (OS) as the primary outcome, which was defined as the duration between the initial diagnosis and death due to any causes. Additionally, the secondary outcome was to analyze OS separately as two mutually exclusive events: (1) death specifically resulting from papillary thyroid carcinoma (CSS); and (2) death occurring due to any other causes (OC) unrelated to papillary thyroid carcinoma.

### Cox proportional hazard survival analysis

This study utilized univariate Cox proportional hazards regression and Kaplan–Meier curves to identify predictors of patient outcomes. Variables that were found to be significant in the univariate analysis (*p* < 0.05) were included in a multivariate Cox proportional hazards model. The most predictive model was chosen using a backward selection process (entry criterion: *p* < 0.05, elimination criterion: *p* > 0.10). Finally, multivariate Cox proportional hazards regression was used to identify variables that had a significant impact on the OS of the PTC patients in the study cohort. The analysis method can be referred to another article we published earlier (26).

### Competing risk analysis of overall deaths

In the competing risk model, deaths from other causes (OC) were regarded as a competing event for PTC-specific death. We first computed the cumulative incidence function (CIF) for PTC and OC. The significant differences in CIF values among subgroups were evaluated by Gray’s test (27). A stepwise competing risk regression model was developed by identifying variables that were significant in the univariate CIF analysis with a *p*-value of less than 0.1. The optimal regression model was constructed by integrating the predicted variables obtained from the stepwise regression procedure. We then estimated the sub-distribution hazard ratio (SHR) for patients diagnosed with PTC using a multivariate competing risk model fitted with the R package “riskRegression.”

### Multinomial logistic regression analysis

A multinomial logistic regression model could classify a patient into one of three possible time-to-surgery groups: ≤3 months; >3 months and ≤6 months; and > 6 months. The final multinomial model included age, gender, race, marital status at diagnosis, median household income, and rural–urban dwelling environmental setting as factors reflecting a patient’s sociodemographic attributes. The R package “nnet” (28) was used to perform the logistic regression.

### Statistical analysis

We presented descriptive statistics in Table 1 for the entire study cohort and compared the results across time-to-surgery subgroups. Continuous and categorical variables were assessed with the Kruskal-Wallis test and Pearson chi-square test, respectively. Continuous variables were expressed as the mean ± standard deviation (SD)/median. All statistical analyses were carried out employing the R studio version 4.0.4. A two-tailed *p* < 0.05 was considered statistically significant.

**Table 1:** Demographic, socioeconomic, and clinical characteristics by time-to-surgery groups in papillary thyroid cancer patients.

		Time-to-surgery			
	Total	≤3 months	3–6 months	>6 months	*P*-value
N	58,378	52,975	4,148	1,255	
Age, Mean ± SD	48.0 ± 15.5	47.8 ± 15.4	50.3 ± 15.9	49.5 ± 16.5	<0.001
Sex, N (%)					0.706
Female	44,156 (75.6%)	40,087 (75.7%)	3,116 (75.1%)	953 (75.9%)	
Male	14,222 (24.4%)	12,888 (24.3%)	1,032 (24.9%)	302 (24.1%)	
Race, N (%)					<0.001
White	46,928 (80.4%)	42,804 (80.8%)	3,179 (76.6%)	945 (75.3%)	
Black	2,292 (3.9%)	2022 (3.8%)	206 (5.0%)	64 (5.1%)	
^a^Others	9,158 (15.7%)	8,149 (15.4%)	763 (18.4%)	246 (19.6%)	
Pathology, N (%)					0.091
C-PTC	46,396 (79.5%)	42,141 (79.5%)	3,288 (79.3%)	967 (77.1%)	
FV-PTC	11,982 (20.5%)	10,834 (20.5%)	860 (20.7%)	288 (22.9%)	
SEER stage, N (%)					<0.001
Localized	21,207 (36.3%)	19,357 (36.5%)	1,410 (34.0%)	440 (35.1%)	
Regional	19,405 (33.2%)	17,962 (33.9%)	1,112 (26.8%)	331 (26.4%)	
Unstaged	17,766 (30.4%)	15,656 (29.6%)	1,626 (39.2%)	484 (38.6%)	
Tumor size categorical, N (%)					<0.001
≤1 cm	32,934 (56.4%)	29,581 (55.8%)	2,582 (62.2%)	771 (61.4%)	
>1 cm and ≤2 cm	13,786 (23.6%)	12,634 (23.8%)	886 (21.4%)	266 (21.2%)	
>2 cm and ≤3 cm	6,391 (10.9%)	5,912 (11.2%)	374 (9.0%)	105 (8.4%)	
>3 cm and ≤4 cm	2,698 (4.6%)	2,500 (4.7%)	143 (3.4%)	55 (4.4%)	
>4 cm and ≤5 cm	1,436 (2.5%)	1,315 (2.5%)	92 (2.2%)	29 (2.3%)	
>5 cm	1,133 (1.9%)	1,033 (1.9%)	71 (1.7%)	29 (2.3%)	
Tumor extension, N (%)					<0.001
Within thyroid capsule	27,037 (46.3%)	24,735 (46.7%)	1774 (42.8%)	528 (42.1%)	
T3b	6,834 (11.7%)	6,289 (11.9%)	411 (9.9%)	134 (10.7%)	
T4a	916 (1.6%)	846 (1.6%)	61 (1.5%)	9 (0.7%)	
T4b	305 (0.5%)	283 (0.5%)	17 (0.4%)	5 (0.4%)	
Unspecified	23,286 (39.9%)	20,822 (39.3%)	1885 (45.4%)	579 (46.1%)	
Lymph node involvement, N (%)					<0.001
N0	23,611 (40.4%)	21,526 (40.6%)	1,606 (38.7%)	479 (38.2%)	
N1a	8,518 (14.6%)	7,882 (14.9%)	497 (12.0%)	139 (11.1%)	
N1b	3,351 (5.7%)	3,102 (5.9%)	185 (4.5%)	64 (5.1%)	
Unspecified	22,898 (39.2%)	20,465 (38.6%)	1860 (44.8%)	573 (45.7%)	
Surgery, N (%)					<0.001
Lobectomy	4,810 (8.2%)	4,055 (7.7%)	572 (13.8%)	183 (14.6%)	
Total thyroidectomy	53,568 (91.8%)	48,920 (92.3%)	3,576 (86.2%)	1,072 (85.4%)	
Neck dissection, N (%)					<0.001
No	18,232 (31.2%)	16,243 (30.7%)	1,499 (36.1%)	490 (39.0%)	
Yes	36,524 (62.6%)	33,338 (62.9%)	2,481 (59.8%)	705 (56.2%)	
Unspecified	3,622 (6.2%)	3,394 (6.4%)	168 (4.1%)	60 (4.8%)	
RAI, N (%)					<0.001
No/Unknown	27,318 (46.8%)	23,946 (45.2%)	2,527 (60.9%)	845 (67.3%)	
Yes	31,060 (53.2%)	29,029 (54.8%)	1,621 (39.1%)	410 (32.7%)	
Marital status at diagnosis, N (%)					<0.001
Single	19,954 (34.2%)	17,835 (33.7%)	1,638 (39.5%)	481 (38.3%)	
Married	35,730 (61.2%)	32,758 (61.8%)	2,278 (54.9%)	694 (55.3%)	
Unknown	2,694 (4.6%)	2,382 (4.5%)	232 (5.6%)	80 (6.4%)	
Median household income, N (%)					0.002
“$35,000–$64,999”	20,310 (34.8%)	18,466 (34.9%)	1,391 (33.5%)	453 (36.1%)	
“$65,000–$74,999”	15,189 (26.0%)	13,691 (25.8%)	1,132 (27.3%)	366 (29.2%)	
“$75,000+”	22,879 (39.2%)	20,818 (39.3%)	1,625 (39.2%)	436 (34.7%)	
Residential setting, N (%)					<0.001
^b^Rural	3,871 (6.6%)	3,570 (6.7%)	217 (5.2%)	84 (6.7%)	
^c^Urban	54,507 (93.4%)	49,405 (93.3%)	3,931 (94.8%)	1,171 (93.3%)	
Cause of death, N (%)					<0.001
Censored	54,656 (93.6%)	49,722 (93.9%)	3,806 (91.8%)	1,128 (89.9%)	
PTC	672 (1.2%)	607 (1.1%)	48 (1.2%)	17 (1.4%)	
Other causes	3,050 (5.2%)	2,646 (5.0%)	294 (7.1%)	110 (8.8%)	
Survival months, Mean ± SD/Median	85.2 ± 61.7/74.0	86.7 ± 62.0/76.0	70.1 ± 56.9/54.5	72.1 ± 58.4/55.0	<0.001

a Others, American Indian/Alaska Native, Asian/Pacific Islander; C-PTC, classic papillary thyroid cancer; FV-PTC, follicular variant papillary thyroid cancer; SEER, the Surveillance, Epidemiology, and End Results program; SEER stage: see materials and methods. RAI, radioactive iodine.

b Rural, population < 250,000.

c Urban, population ≥ 250,000.

### Institutional review board waiver statement

No institutional review board approval was required since SEER is an open-access public database with a deidentified dataset.

## Results

**Figure 2:**
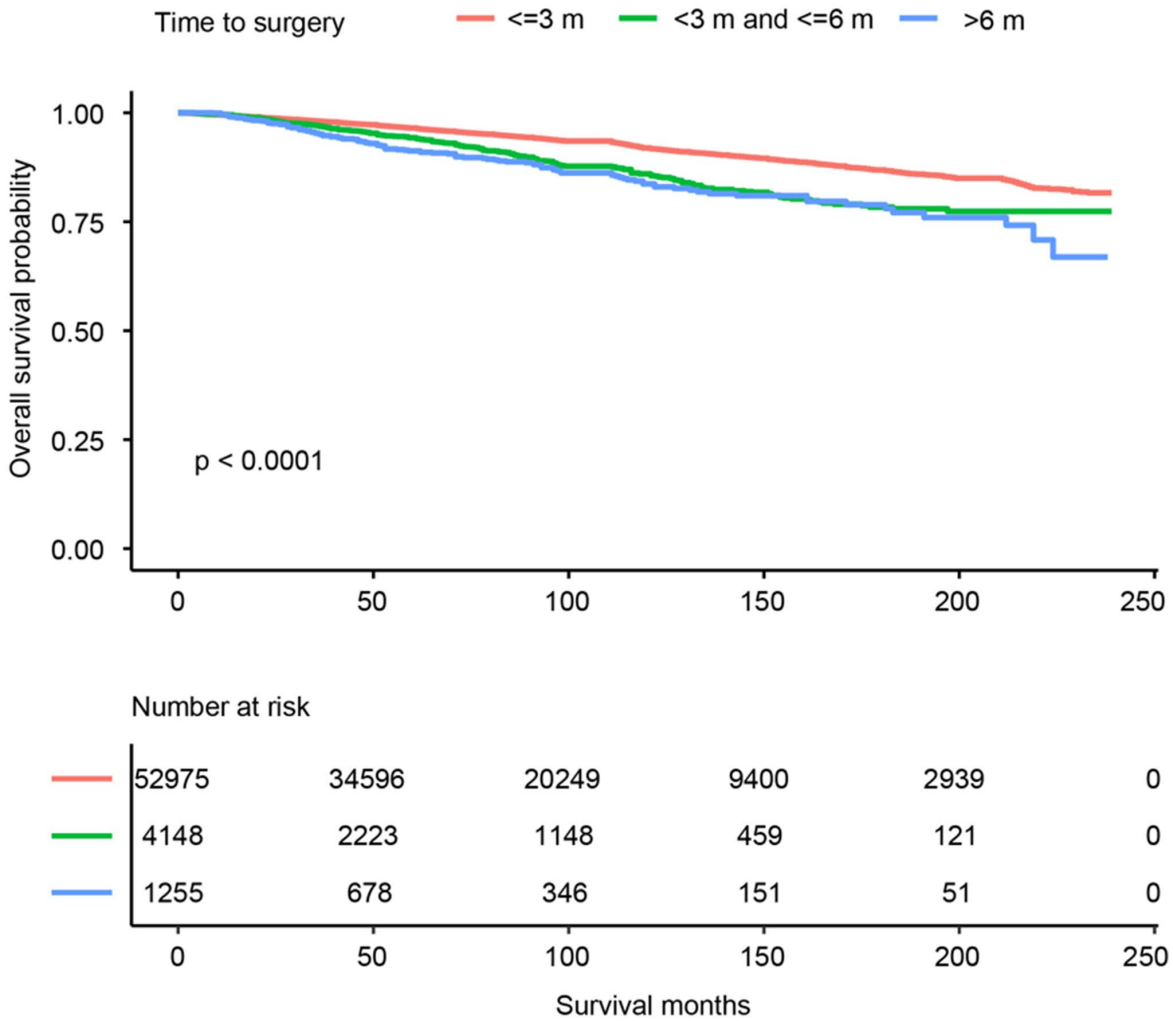
Kaplan–Meier survival curves for overall survival.

A total of 58,378 PTC patients were studied. This group of patients was typically middle-aged (average age of 48.0 years, ranging from 32.5 to 63.5), predominantly white (46,928, 80.4%), with a female to male prevalence of 3:1, and more than 60% married at diagnosis. They mostly resided in urban areas (93.4%) and had a household income that was evenly distributed across the low to high range. The median follow-up of the study cohort was 74 months (range from 1 month to 239 months). The total number of deaths during the follow-up period was 3,722 (6.4%), of which 672 (1.2%) were attributed to PTC and 3,050 (5.2%) to non-PTC causes, thus rendering a primarily non-cancer death pattern. The OS trend was plotted in Figure 2. Overall, the 5- and 10-year OS was 96.1% (CI: 95.9–96.3%) and 91.2% (CI: 90.9–91.6%), respectively. More SES factors associated with 5- and 10-year survival rates are detailed in Table 2.

**Table 2:** Five-year and 10-year overall survival for papillary thyroid carcinoma patients with different socioeconomic factors.

	5-year overall survival		10-year overall survival	
	Overall survival	95% CI	Overall survival	95% CI
Time to surgery, months				
≤3	96.4%	96.2–96.6%	91.8%	91.4–92.1%
>3 and ≤6	94.0%	93.2–94.9%	85.6%	83.9–87.2%
>6	91.3%	89.4–93.2%	83.7%	80.7–86.8%
Marital status at diagnosis				
Single	95.2%	94.9–95.5%	89.2%	88.7–89.8%
Married	96.6%	96.4–96.8%	92.3%	92.0–92.7%
Unknown	96.0%	95.3–96.6%	90.9%	89.5–92.3%
Median household income				
“$35,000–$64,999”	95.6%	95.3–95.8%	89.9%	89.4–90.5%
“$65,000–$74,999”	96.1%	95.9–96.4%	91.2%	90.7–91.8%
“$75,000+”	96.7%	96.5–96.9%	92.4%	92.0–92.8%
Residential setting				
^a^Rural	94.0%	93.3–94.6%	86.5%	85.3–87.8%
^b^Urban	96.3%	96.1–96.5%	91.6%	91.3–91.9%

PTC, papillary thyroid cancer.

a Rural, population < 250,000.

b Urban, population ≥ 250,000.

The study cohort was divided into three groups based on the length of their surgery delay. The majority of patients (90.7%) received thyroid surgery within 3 months, while a smaller percentage (7.1%) received surgery between 3 and 6 months. Only 2.2% of the cohort had to wait more than 6 months to undergo thyroid surgery. There are noticeable disparities in the clinicopathological and sociodemographic characteristics across the groups, except for the gender ratio, which was found to be statistically the same in all groups (*p* = 0.706). Furthermore, the tumors observed among such individuals are typically smaller—less than 1 cm. It was observed that this group tends to undergo lobectomy with higher frequency and showed a lower probability of receiving radioactive iodine (RAI) therapy. Additionally, married individuals are less likely to be a part of this category. This data is outlined in Table 1.

In the multivariate Cox analysis for OS, advanced age (e.g., age > 45 and ≤55 vs. age ≤45, HR = 3.12, 95% CI 2.73–3.57, *p* < 0.001) was estimated as the leading independent prognostic factors associated with worse OS outcomes. The other independent prognostic factors, in descending order of significance, were more invasive tumor extension (T4a compared to confined to thyroid capsule, HR = 1.99, 95% CI 1.71–2.31, *p* < 0.001), larger tumor size (>5 cm vs. ≤1 cm, HR = 1.77, 95% CI 1.23–2.53, *p* = 0.002), advanced AJCC N stage (N1b compared to N0, HR = 1.76, 95% CI 1.53–2.01, *p* < 0.001), longer surgery delay (>6 months vs. ≤3 months, HR = 1.73, 95% CI 1.44–2.06, *p* < 0.001), and Black ethnicity (vs. white, HR = 1.18, 95% CI 1.02–1.37, *p* = 0.028), which was a borderline significant predictor after adjustment and model selection from the univariate Cox regression. Receiving neck dissection (vs. not performed, HR = 0.78, 95% CI 0.72–0.85, *p* < 0.001), radioactive iodine therapy (RAI) (vs. not performed, HR = 0.76, 95% CI 0.71–0.81, *p* < 0.001), being married at diagnosis (vs. single, HR = 0.72, 95% CI 0.67–0.77, *p* < 0.001), living in an urban area (vs. rural, HR = 0.77, 95% CI 0.69–0.86, *p* < 0.001), having a medium to high income (median household income: “$75,000+” vs. “$35,000 - $64,999,” HR = 0.83, 95% CI 0.77–0.90, *p* < 0.001), and being of American Indian/Alaska Native, Asian/Pacific Islander ethnicity (vs. white, HR = 0.83, 95% CI 0.75–0.92, *p* < 0.001) were estimated to be the independent predictors for better OS. The pathological classification of the lesion (follicular variant vs. classic-PTC, *p* = 0.118) and the chosen surgical procedure (*p* = 0.954) were found to have no bearing on the OS rate. The Cox regression results are presented in Table 3.

**Table 3:** Univariate and multivariate Cox proportional hazard regression for analyses of papillary thyroid carcinoma patients for overall survival.

	Multivariate			Univariate		
	HR	95% CI	*p*-value	HR	95% CI	*p*-value
Time to surgery, months						
≤3	1	Reference		1	Reference	
>3 and ≤6	1.39	(1.24–1.56)	<0.001	1.72	(1.54–1.92)	<0.001
>6	1.73	(1.44–2.06)	<0.001	2.04	(1.71–2.44)	<0.001
Marital status at diagnosis						
Single	1	Reference		1	Reference	
Married	0.72	(0.67–0.77)	<0.001	0.70	(0.66–0.75)	<0.001
Unknown	0.83	(0.70–0.98)	0.031	0.84	(0.70–0.99)	0.038
Median household income						
“$35,000–$64,999”	1	Reference		1	Reference	
“$65,000–$74,999”	0.96	(0.88–1.05)	0.345	0.87	(0.80–0.94)	<0.001
“$75,000+”	0.83	(0.77–0.90)	<0.001	0.75	(0.69–0.80)	<0.001
Residential setting						
^b^Rural	1	Reference		1	Reference	
^c^Urban	0.77	(0.69–0.86)	<0.001	0.61	(0.55–0.68)	<0.001
Race						
White	1	Reference		1	Reference	
Black	1.18	(1.02–1.37)	0.028	1.23	(1.06–1.42)	0.007
^a^Others	0.83	(0.75–0.92)	0.001	0.78	(0.71–0.87)	<0.001
Age						
≤45	1	Reference		1	Reference	
>45 and ≤55	3.12	(2.73–3.57)	<0.001	2.91	(2.55–3.32)	<0.001
>55 and ≤65	7.50	(6.63–8.48)	<0.001	7.30	(6.46–8.24)	<0.001
>65	22.49	(20.08–25.20)	<0.001	23.54	(21.05–26.32)	<0.001
Pathology						
C-PTC	1	Reference		1	Reference	
FV-PTC	0.94	(0.87–1.02)	0.118	1.15	(1.07–1.24)	<0.001
Tumor size						
≤1 cm	1	Reference		1	Reference	
>1 cm and ≤2 cm	1.04	(0.94–1.14)	0.486	0.90	(0.83–0.98)	0.016
>2 cm and ≤3 cm	1.13	(1.00–1.27)	0.044	1.02	(0.92–1.13)	0.700
>3 cm and ≤4 cm	1.50	(1.30–1.73)	<0.001	1.41	(1.24–1.61)	<0.001
>4 cm and ≤5 cm	1.46	(1.22–1.74)	<0.001	1.56	(1.32–1.83)	<0.001
>5 cm	1.97	(1.66–2.34)	<0.001	2.39	(2.05–2.80)	<0.001
Tumor extension						
Confined to capsule	1	Reference		1	Reference	
T3b	1.29	(1.17–1.42)	<0.001	1.50	(1.37–1.65)	<0.001
T4a	1.99	(1.71–2.31)	<0.001	3.48	(3.01–4.02)	<0.001
T4b	1.95	(1.52–2.50)	<0.001	3.30	(2.59–4.19)	<0.001
Unspecified	1.26	(1.02–1.57)	0.034	1.22	(1.12–1.32)	<0.001
Lymph node involvement						
N0	1	Reference		1	Reference	
N1a	1.21	(1.08–1.36)	0.001	0.77	(0.69–0.85)	<0.001
N1b	1.76	(1.53–2.01)	<0.001	1.32	(1.17–1.48)	<0.001
Unspecified	1.16	(0.93–1.44)	0.193	1.00	(0.92–1.08)	0.930
Surgery						
Lobectomy	1	Reference		1	Reference	
Total thyroidectomy	1.00	(0.89–1.13)	0.954	0.81	(0.72–0.91)	<0.001
Neck dissection						
No	1	Reference		1	Reference	
Yes	0.78	(0.72–0.85)	<0.001	0.67	(0.62–0.72)	<0.001
Unspecified	0.93	(0.81–1.07)	0.299	0.74	(0.67–0.83)	<0.001
RAI						
No/Unknown	1	Reference		1	Reference	
Yes	0.76	(0.71–0.81)	<0.001	0.75	(0.70–0.80)	<0.001

HR, hazard ratio; CI, confidential interval.

a Others, American Indian/Alaska Native, Asian/Pacific Islander; C-PTC, classic papillary thyroid cancer; FV-PTC, follicular variant papillary thyroid cancer; SEER, the Surveillance, Epidemiology, and End Results program; SEER stage: see materials and methods; RAI, radioactive iodine.

b Rural, population < 250,000.

c Urban, population ≥ 250,000.

**Figure 3:**
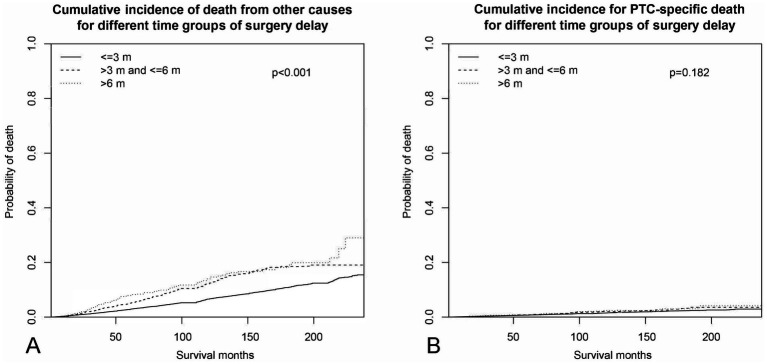
Cumulative incidence curves across different periods of surgery delay. **(A)** Cumulative incidence of non-PTC deaths. **(B)** Cumulative incidence of PTC-specific deaths.

Results of the competing risk of death hazards regression analysis of sociodemographic factors were estimated and shown in Table 4. Age was excluded from the analysis, as it is known to be the predominant factor for mortality for each competing party. The CIF of different lengths of surgery delay for PTC and OC is illustrated in Figure 3. Delaying surgery for a longer duration was found to be a significant predictor of higher death rates from non-PTC causes (e.g., >6 months vs. ≤3 months: Non PTC-specific SHR = 1.65, 95% CI 1.32–2.07, *p* < 0.001). However, this delay in surgery did not represent a significant risk factor for CSS (e.g., >6 months vs. ≤3 months: PTC-specific SHR = 1.01, 95% CI 0.58–1.77, *p* = 0.996). Being male is the only factor that is strongly associated with a higher risk of mortality from both non-PTC causes (vs. female: SHR = 1.96, 95% CI 1.80–2.12, *p* < 0.001) and PTC-specific deaths (vs. female: PTC-specific SHR = 2.24, 95% CI 1.91–2.62, *p* < 0.001). Racial ethnicity is not a significant predictor of CSS (black vs. white: SHR = 1.00, 95% CI 0.68–1.49, *p* = 0.984, others vs. white: SHR = 1.01, 95% CI 0.79–1.31, *p* = 0.914). However, being of other minority ethnicities (American Indian/Alaska Native or Asian/Pacific Islander ethnicities) was associated with a 34% decrease in mortality from non-PTC causes (vs. white: SHR = 0.66, 95% CI 0.54–0.81, *p* < 0.001), while there was a 21% increase in mortality risk for Black compared to white ethnicity (vs. white: SHR = 1.21, 95% CI 1.00–1.46, *p* = 0.048). Married PTC patients were inclined to have a lower non-PTC death rate compared to single patients at the time of diagnosis (married vs. single: SHR = 0.82, 95% CI 0.75–0.89, *p* < 0.001). Yet, marital status showed no predictive value in PTC-specific death risk (married vs. single: SHR = 0.92, 95% CI 0.77–1.09, *p* = 0.318). Higher household yearly income was estimated to be the only significant SES factor that predicted a lower CSS (e.g., “$75,000+” vs. “$35,000–$64,999”: SHR = 0.73, 95% CI 0.59–0.90, *p* = 0.004), as opposed to those with lower incomes. However, this same factor was not predictive of non-cancer related mortality (e.g., “$75,000+” vs. “$35,000–$64,999”: SHR = 0.92, 95% CI 0.78–1.07, *p* = 0.270). Moreover, living in urban areas was found to be associated with a lower risk of non-cancer related mortality compared to living in rural areas (urban vs. rural: SHR = 0.61, 95% CI 0.51–0.72, *p* < 0.001). However, this difference was not observed in terms of CSS (urban vs. rural: SHR = 0.93, 95% CI 0.69–1.24, *p* = 0.608).

**Table 4:** Competing risk hazard regression of survival for sociodemographic factors in papillary thyroid carcinoma patients.

	Death from PTC			Death from OC		
	SHR	95% CI	*P*-value	SHR	95% CI	*P*-value
Sex						
Female	1	Reference		1	Reference	
Male	2.24	(1.91–2.62)	<0.001	1.96	(1.80–2.12)	<0.001
Race						
White	1	Reference		1	Reference	
Black	1.00	(0.68–1.49)	0.984	1.21	(1.00–1.46)	0.048
^a^Others	1.01	(0.79–1.31)	0.914	0.66	(0.54–0.81)	<0.001
Time to surgery, months						
≤3	1	Reference		1	Reference	
>3 and ≤6	1.03	(0.74–1.43)	0.855	1.35	(1.17–1.57)	<0.001
>6	1.01	(0.58–1.77)	0.966	1.65	(1.32–2.07)	<0.001
Marital status at diagnosis						
Single	1	Reference		1	Reference	
Married	0.92	(0.77–1.09)	0.318	0.82	(0.75–0.89)	<0.001
Unknown	0.91	(0.59–1.40)	0.669	0.71	(0.57–0.88)	0.002
Median household income						
“$35,000–$64,999”	1	Reference		1	Reference	
“$65,000–$74,999”	0.72	(0.56–0.91)	0.007	0.96	(0.82–1.11)	0.554
“$75,000+”	0.73	(0.59–0.90)	0.004	0.92	(0.78–1.07)	0.270
Residential setting						
^b^Rural	1	Reference		1	Reference	
^c^Urban	0.93	(0.69–1.24)	0.608	0.61	(0.51–0.72)	<0.001

PTC, papillary thyroid cancer; OC, other causes other than PTC; SHR, sub-distribution hazard ratio; CI, confidential interval.

a Others, American Indian/Alaska Native, Asian/Pacific Islander.

b Rural, population < 250,000.

c Urban, population ≥ 250,000.

Table 5 showed the adjusted results of multinomial logistic regression estimates between sociodemographic factors and duration of surgery delay. The unmarried (married vs. single: OR = 0.78, 95% CI 0.69–0.88, *p* < 0.001), older (e.g., >55 and ≤65 vs. ≤45: OR = 1.23, 95% CI 1.05–1.44, *p* = 0.009), minority ethnicity (e.g., others vs. white: OR = 1.45, 95% CI 1.25–1.67, *p* < 0.001) population was independently associated with longer delays (both >6 months and 3 to 6 months), compared to less than 3 months of surgery delay. It was found that the various income levels of households were not able to distinguish PTC patients who had a time-to-surgery of less than 3 months from those who had a time-to-surgery of 3–6 months, independently. However, the population earning an annual income exceeding $75,000 were less inclined to wait for surgery beyond 6 months, as opposed to those who waited for less than 3 months (“$75,000+” vs. “$35,000–$64,999”: OR = 0.84, 95% CI 0.73–0.96, *p* = 0.012). Residents in urban areas, as opposed to those in rural areas, were found to have a significant correlation with patients who underwent PTC surgery within 3–6 months (vs. <3 months: OR = 1.28, 95% CI 1.10–1.48, *p* = 0.001), rather than those who experienced a surgery delay of over 6 months (vs. <3 months: OR = 1.02, 95% CI 0.81–1.29, *p* = 0.878).

**Table 5:** Multinomial logistic regression analysis of socioeconomic and demographic factors across different surgery delay groups.

	Surgery delay, months					
	>3 and ≤6 vs. ≤3			>6 vs. ≤3		
	OR	95% CI	*P*-value	OR	95% CI	*P*-value
Sex						
Female	1	Reference		1	Reference	
Male	1.02	(0.95–1.10)	0.523	0.99	(0.86–1.12)	0.827
Race						
White	1	reference		1	reference	
Black	1.29	(1.11–1.49)	0.001	1.34	(1.03–1.74)	0.026
^a^Others	1.29	(1.19–1.40)	<0.001	1.45	(1.25–1.67)	<0.001
Age						
≤45	1	Reference		1	Reference	
>45 and ≤55	1.15	(1.06–1.26)	0.001	1.09	(0.94–1.26)	0.267
>55 and ≤65	1.41	(1.29–1.53)	<0.001	1.23	(1.05–1.44)	0.009
>65	1.53	(1.40–1.68)	<0.001	1.52	(1.30–1.79)	<0.001
Marital status at diagnosis						
Single	1	Reference		1	Reference	
Married	0.74	(0.69–0.79)	<0.001	0.78	(0.69–0.88)	<0.001
Unknown	1.04	(0.90–1.20)	0.571	1.24	(0.97–1.58)	0.082
Median household income						
“$35,000–$64,999”	1	Reference		1	Reference	
“$65,000–$74,999”	1.07	(0.98–1.16)	0.124	1.09	(0.95–1.26)	0.231
“$75,000+”	1.00	(0.92–1.08)	0.978	0.84	(0.73–0.96)	0.012
Residential setting						
^b^Rural	1	Reference		1	Reference	
^c^Urban	1.28	(1.10–1.48)	0.001	1.02	(0.81–1.29)	0.878

OR, odds ratio; CI, confidential interval.

a Others, American Indian/Alaska Native, Asian/Pacific Islander.

b Rural, population < 250–000.

c Urban, population ≥250,000.

## Discussion

Delaying surgery for non-metastatic PTC patients resulted in a lower OS rate. This finding is in agreement with a large-scale National Cancer Database (NCDB) study (16). As we categorized the mortality rate into two mutual exclusive causes of death—PTC-specific and OC—in the competing risk of death regression analysis, we found that surgery delay was not a significant predictor of the PTC-specific survival rate (>3 and ≤6 vs. ≤3, *p* = 0.855; >6 vs. ≤3, *p* = 0.966), as shown in Figure 3B. This implies that the reduced OS rate observed was not relevant to PTC-specific deaths. Our curiosity was quickly drawn by the question of which factors influence the relationship between delaying surgery and an increase in OC-associated mortality.

To understand the relationship between surgery delay and increased mortality rates associated with OC, we initially removed the impact of PTC-related factors by definition. According to the results in Table 4, the unmarried Black male who lives in a rural area with a longer surgery delay is more likely to suffer from higher non-PTC mortality. This finding is consistent with previous studies (12, 14, 29) that have suggested an association between lower SES and poorer survival outcomes among PTC patients. It is important to consider whether the population that experienced a longer surgery delay may also be the same population with lower SES.

To clarify this, a multinomial logistic regression was conducted as a population classifier to identify the association between SES factors and different lengths of surgery delay. The group of PTC patients who tended to experience a longer surgery delay in general was the unmarried older minority ethnicities (Table 5). Gender was not relevant to longer surgery delay. High income (yearly income higher than $75,000) patients were more likely to receive timely surgery. Urban dwellers were more likely to experience a moderate period of surgery delay (3–6 months) as opposed to those living in rural areas. This could be interpreted that high-quality medical resource can be more centralized in metropolis, so as population density.

There are several noteworthy findings that need to be highlighted. Even after adjusting for all clinicopathological and sociodemographic factors, the current study with 58,378 non-metastatic PTC patients found that delaying surgery was still a significant predictor of lower OS in non-metastatic PTC patients. This suggests a strong and consistent relationship between surgery delay and OS, which is consistent with several previous studies (16, 30). During the univariate Cox hazard regression analysis, thyroid surgery appeared to be a significant predictor of increased OS. However, after adjusting for various factors in the multivariate Cox analysis, the type of surgery, whether total thyroidectomy or lobectomy, became irrelevant (HR = 1.00, 95% CI 0.89–1.13, *p* = 0.954). This suggests that the method of surgery is not an independent predictor of OS. In patients with PTC, those of Black ethnicity have a 23% higher chance of overall mortality than those of white. The reasons may be partly attributed to socioeconomic factors and cultural differences. With all results and deductions summarized above, the authors came up with a hypothesis that in the context of a pre-end stage malignancy with low mortality rate, age was the ranking survival predictor, followed by other TNM and medical care related factors and SES factors. Patients with a longer surgery delay highly overlapped with the population with a low SES, indicating the interrelationship of these factors.

Our study has several limitations that must be acknowledged. First, the imbalanced sample sizes across surgery delay groups may have impacted the statistical power and accuracy of our findings. Selection bias is another significant limitation, stemming from the data registry’s exclusion criteria and the retrospective nature of the study. For instance, excluding 96,030 patients who underwent immediate surgery may overestimate the average time to surgery and skew the representation of patient outcomes. Additionally, unadjusted confounding variables, such as comorbidities and healthcare access, could influence both the time to surgery and survival outcomes, potentially distorting our conclusions. The retrospective design further limits our ability to establish causal relationships and control for unmeasured confounders.

While the SEER database offers a robust sample, its coverage of only about 10% of the U.S. population, focused on specific regions, limits the generalizability of our findings to the broader population. These biases primarily affect internal validity by potentially overestimating or underestimating the associations observed and external validity by reducing applicability to non-SEER-covered regions. Therefore, our results should be interpreted with caution, as they may reflect the sociodemographic and healthcare characteristics unique to SEER-covered areas.

Our study also provides insights into the importance of considering socioeconomic factors and surgery delay when managing PTC patients. Those from low SES backgrounds may have pre-existing medical conditions and limited access to healthcare, which may increase their risk of non-PTC mortality. The current study may serve as a piece of evidence for health care professionals, policymakers, and the general public as they strive to further medical treatment and research, make informed decisions, and improve survival outcomes.

## Conclusion

This study provides valuable insights into the relationship between surgery delay, SES, and survival outcomes in non-metastatic papillary thyroid cancer (PTC) patients. Our findings suggest that surgery for non-metastatic PTC can be safely delayed without significantly increasing PTC-specific mortality, emphasizing the importance of individualized treatment plans. However, the elevated non-PTC mortality observed in populations with lower SES highlights disparities in access to healthcare and broader socioeconomic inequities. Independent predictors of better overall survival, such as receiving neck dissection or radioactive iodine therapy, being married, and living in urban, higher-income areas, further underscore the interplay of sociodemographic and clinical factors.

These findings call for targeted interventions to address healthcare disparities, particularly for vulnerable populations, including older, unmarried, rural, or minority patients. Future research should explore strategies to mitigate these disparities and further investigate the nuanced effects of SES and treatment timing on survival outcomes.

## Data Availability

The original contributions presented in the study are included in the article/supplementary material, further inquiries can be directed to the corresponding authors.
